# Platelet Bone Morphogenetic Protein-4 Mediates Vascular Inflammation and Neointima Formation after Arterial Injury

**DOI:** 10.3390/cells10082027

**Published:** 2021-08-08

**Authors:** Marietta Jank, Nikolaus von Niessen, Christoph B. Olivier, Hannah Schmitt, Nathaly Anto-Michel, Ingo Hilgendorf, Christoph Bode, Martin Moser, Jennifer S. Esser, Qian Zhou

**Affiliations:** 1 University Heart Center Freiburg—Bad Krozingen, Department of Cardiology and Angiology I, Faculty of Medicine, University of Freiburg, 79106 Freiburg, Germany; Marietta.Jank@umm.de (M.J.); nikolaus.von.niessen@gmail.com (N.v.N.); christoph.olivier@universitaets-herzzentrum.de (C.B.O.); hannah.schmitt@universitaets-herzzentrum.de (H.S.); nathaly.anto-michel@medunigraz.at (N.A.-M.); ingo.hilgendorf@universitaets-herzzentrum.de (I.H.); christoph.bode@universitaets-herzzentrum.de (C.B.); martin.moser@universitaets-herzzentrum.de (M.M.); jennifer.esser@universitaets-herzzentrum.de (J.S.E.); 2 University Heart Center Freiburg—Bad Krozingen, Institute for Experimental Cardiovascular Medicine, Faculty of Medicine, University of Freiburg, 79106 Freiburg, Germany; 3 Department of Medicine, Division of Cardiology, University Hospital Basel, 4031 Basel, Switzerland

**Keywords:** BMP, platelet, restenosis, inflammation

## Abstract

The purpose of this study is to investigate the role of platelet bone morphogenetic proteins (BMP)-4 during vascular inflammation and remodeling in a mouse model of carotid wire injury. Transgenic mice with a platelet-specific deletion of BMP-4 (*BMP4^Plt−/−^*) were generated. Intravital microscopy was performed to evaluate leukocyte adhesion to the vessel wall. Expression of adhesion molecules and chemokines were analyzed. Platelet-leukocyte aggregates (PLAs) were evaluated using flow cytometry. For carotid wire injury, *BMP4^Plt^*^−/−^ mice were further crossed with LDLr^−/−^ mice (*BMP4^Plt−/−^/LDLr^−/−^*) and fed with a high cholesterol diet for 2-weeks. Carotid wire injury was performed, and re-endothelialization and neointimal formation were evaluated. In comparison to the control mice, stimulation with TNFα resulted in fewer rolling and adherent leukocytes to the vessel wall in the *BMP4^Plt−/−^* mice. mRNA and protein expression of P-selectin and adhesion molecules were reduced in the aorta of the *BMP4^Plt−/−^* mice. In platelets from the *BMP4^Plt−/−^* mice, the expression of P-selectin was reduced, and fewer PLA formations were measured than in the control mice. Loss of platelet BMP-4 further prevented neointima formation after carotid wire injury. Endothelial regeneration after injury was decelerated in the *BMP4^Plt−/−^* mice, and confirmed in-vitro, where the deletion of platelet BMP-4 inhibited endothelial cell proliferation and migration. We demonstrate for the first time that platelet BMP-4 is involved during vascular inflammation and remodeling. This is partially mediated by the inhibition of platelet activation, reduced expression of adhesion molecules and inflammatory responses. Our findings identify platelet BMP-4 as a mediator of vascular inflammation in early atherosclerosis and restenosis.

## 1. Introduction

Atherosclerosis is a chronic inflammatory response to injury of the vessel wall [1]. It is an interplay of various cell-types, including leukocytes, platelets, smooth muscle cells and endothelial cells. While the role of leukocytes in mediating inflammatory processes has been well characterized over the past years, the role of platelets in this process is still not fully understood. For a long time, the activation of platelets at sites of vascular injury has only been linked to thrombosis and hemostasis. However, emerging data in the past years suggest a pivotal role of platelets in inflammatory reactions. Indeed, platelets have been shown to play a significant role in various inflammatory diseases, including atherosclerosis, bacterial or viral infections, and acute lung injury [2]. A key function of platelets during the inflammatory response is its interplay with leukocytes. This is mainly mediated by P-selectin, its ligand P-selectin glycoprotein ligand (PSGL)-1 [3], and various chemokines, including regulated on activation, normal T cell expressed and secreted (RANTES, CCL5) and platelet factor-4 (PF-4, CXCL4), which trigger activation and adhesion of inflammatory cells to the vascular wall [4,5], but have further shown an additive effect on neutrophils and monocytes [6].

Bone morphogenetic proteins (BMPs) are members of the transforming growth factor-β (TGF β) superfamily. BMPs were first identified by their ability to induce ectopic bone formation. Meanwhile, various studies have demonstrated that BMPs are multifunctional regulators in development, hemostasis and disease [7]. BMPs exert their signals via type I and type II serine/ threonine kinase receptors. Upon activation by a ligand, the type II receptor kinase phosphorylates the type I receptor, which initiates the intracellular signaling by activating the Smad proteins. Smads are transcriptions factors and can be divided into three groups: receptor-regulated Smads (R-Smads), inhibitory Smads (I-Smads), and a common-mediator Smad (i.e., Smad-4). Upon activation of the type I receptor, the R-Smads–Smad-1/5/9 are phosphorylated and form heteromeric complexes with Smad-4, which translocate to the nucleus to modulate further gene expression [8,9]. 

Emerging findings suggest a distinct role of BMPs in cardiovascular development and disease. While BMP signaling in endothelial cells is well characterized [10], its role in platelets is poorly understood. Previous studies have demonstrated that megakaryocytes and platelets contain BMP-2, and -4 [11]. However, their role in platelet function and hemostasis remains largely unknown. Accordingly, we aimed to investigate the role of platelet BMP-4 during vascular inflammation and remodeling after wire-induced injury. 

## 2. Materials and Methods

### 2.1. Animals 

Age-matched male littermates (4–8 weeks, all on C57BL/6J background) were used for experiments. To delete BMP-4 in platelets, conditional *BMP4^flox/flox^* mice were bred with transgenic mice expressing the Cre recombinase under the control of the PF-4 promotor [12]. The *PF4^Cre^* mice (*Tg(Pf4-icre)^Q3Rsko/J^*) were obtained from Charles River Laboratories (Sulzfeld, Germany). The *LDLr^−/−^* mice (B6.129S7-Ldlr^tm1Her^) were obtained from The Jackson Laboratory (Bar Harbor, ME, USA). The BMP-4 floxed mice (*BMP4^tm4Blh^*) were generously provided by Brigid Hogan (Duke University, Durham, NC, USA). For all surgeries, the animals were pretreated with buprenorphine (0.1 mg/kg body weight) s.c and anesthetized with 2–5% isoflurane. The animals were maintained in the animal facility at the University Hospital Freiburg. The Standing Committee on Animals at University Freiburg approved all protocols pertaining to the experimentation with animals (G15-152). The animals were maintained in a 22 °C room with a 12 h light/dark cycle and received drinking water ad libitum. The mice were fed regular chow unless otherwise indicated.

### 2.2. Tail Bleeding Assay

The mice were anesthetized and 1cm of the tail tip was cut off. The tails were immersed in 0.9% isotonic saline at 37 °C. The amount of time required for bleeding to stop (defined as no blood flow for 1 min) was measured.

### 2.3. Complete Blood Count

Blood was collected from the retro-orbital plexus into EDTA tubes. Peripheral blood counts were performed using an ADVIA 120/2120 automated hematology analyzer (Bayer Health-Care, Leverkusen, Germany).

### 2.4. Platelet Isolation from Mice

Blood samples from 4 mice were obtained and pooled for platelet isolation. Whole blood was mixed with 100 µL of an acid citrate dextrose buffer (ACD 7X: 3.36 g citric acid, 20.58 g trisodium citrate, 1.47 g dextrose) and 5 µL of heparin (1000 IE/mL). To obtain platelet-rich plasma, 300 µL of Tyrode’s buffer (137 mM NaCl, 0.36 mM Na_2_HPO_4_, 2.7 mM KCl, 12 mM NaHCO_3_, 5 mM HEPES, and 10 mM glucose, 0.35% BSA, pH 7.3) was added and the suspension was centrifuged at 250× *g* for 4 min, after which the supernatant was collected and centrifuged for an additional 4 min at 2200× *g.* The pellet was suspended in 2 mL of Tyrode’s buffer with 1 µL of prostaglandine I_2_ sodium salt (Abcam, Cambridge, UK) and 10 µL heparin (1000 IE/mL, Braun, Melsungen, Germany). After 10 min of incubation the suspension was again centrifuged at 1900× *g* for 3 min. This step was repeated twice. The platelet count was then determined by a Neubauer counting chamber (Marienfeld, Lauda-Königshofen, Germany). 

### 2.5. Intravital Microscopy of Mouse Mesenteric Venules

The mice were pre-treated with murine TNFα (10 ng/g body weight, i.p.) 4 h before surgery. The animals were anaesthetized with ketamine (100 mg/kg) and xylazin (5 mg/kg). Leukocytes were fluorescently labelled in-vivo by retroorbital injection of 50 µL of Rhodamine 6G (1 mg/mL, Sigma-Aldrich, St. Louis, MO, USA). A loop of ileal mesentery was exteriorized through a midline incision in the abdominal wall and placed on a plastic chamber to observe the peri-intestinal microcirculation by intravital microscopy. Leukocyte interactions with the endothelial vessel wall were recorded for 1 min at least in 3 veins/ mouse. Leukocytes stationary for >30 s were defined as adherent and were counted as cells that adhere to 100 µm vessel length. The average diameter of the examined venules was normalized to 100 µm. Leukocyte adhesion was quantified by 2 independent investigators in a blinded way. 

### 2.6. Mouse Model of Carotid Injury

Eight-week-old *BMP4^Plt−/−^/LDLr^−/−^* and *BMP4^Plt+/+^/LDLr^−/−^*mice were fed a high cholesterol diet (15.8% *wt/wt* fat, 1.25% cholesterol; E15749-34 (EF D12108 mod.), Kliba Nafag, Kaiseraugst, Switzerland) starting two weeks prior to wire injury and further two weeks after the intervention. Wire-mediated injury of the vessel wall was performed as described previously [13]. Throughout the procedure, the animals were placed on a temperature-controlled heating pad and anesthetized by inhalation of isoflurane (Abbott, Wiesbaden, Germany). After a ventro-lateral neck incision, the left common carotid artery (LCCA), including its bifurcation in the left internal (LICA) and external carotid artery (LECA), was dissected under a microscope. Surgical sutures (5-0; 6-0) were looped around the proximal LCCA and LICA for temporary disruption of blood flow. Two additional ligatures (6-0) were placed around the LECA and the distal one was tied off. An incision hole was cut in the LECA between the two ligatures. A 0.014-in flexible wire (Cross-IT 200YT, Abbott Vascular, Wetzlar, Germany) was inserted into the LCCA and passed into the vessel lumen 3 times in a rotating motion. After the wire was removed, the proximal ligature of the LECA was tied off. The temporary loops around the LCCA and LICA were opened; hence the blood flow is restored. The surgical incision was closed by skin sutures. At given time points the injured LCCA and the un-injured right common carotid artery (RCCA) were harvested for further experiments. 

### 2.7. Mouse Model of Re-Endothelialization

One, 3 and 5 days after wire injury, the re-endothelialization was evaluated by staining with Evan’s Blue dye. Briefly, 100 μL of 5% Evan’s Blue solution (Sigma-Aldrich, Taufkirchen, Germany) was injected into the retrobulbar sinus and was allowed to circulate for 10 min. After euthanisation, transcardial perfusion with saline water was performed. The LCCA and RCCA were dissected, harvested and fixed with formalin for 5 min. The vessel lumen was cut open longitudinally and embedded in Kayser’s Glycerol (Merck, Darmstadt, Germany). Pictures of en face injured LCCA and un-injured RCCA were taken and the endothelial regeneration was evaluated. The total and blue-stained vessel areas were determined by computer-assisted morphometric analysis with ImageJ. The ratio of the blue-stained denuded area to the total carotid area was determined. The proportion of the denuded vessel wall to the total carotid artery was calculated to take into consideration the variability of the vessel area due to anatomical variation and elasticity of the artery when placing it between the slices. 

### 2.8. Morphometric and Immunofluorescence Analysis 

Excised carotid arteries were embedded in a Tissue-Tek O.C.T. compound (Sakura Finetek Europe, Alphen aan den Rijn, Netherlands) and 10 µm serial cryostat sections were cut starting from the bifurcation towards the aortic arch. The slides were stored at −20 °C for further histological analysis. Sections were fixed in ice cold acetone and subjected to standard hematoxylin and eosin staining. Computer-assisted morphometric analysis with ImageJ (Wayne Rashband, NIH, Bethesda, MD, USA) was used to calculate the lumen and neointima area. Immunofluorescence staining was performed on sections at a distance of 240 µm. The tissue was fixed in ice cold acetone and incubated with primary antibodies overnight at 4 °C. The following primary antibodies were used: anti-Actin α-smooth muscle monoclonal mouse FITC conjugated (Sigma-Aldrich, St. Louis, MO, USA), anti-CD31 rabbit anti-mouse polyclonal (Abcam, Cambridge, UK), anti-Mac3 rat anti-mouse (BD, San Diego, CA, USA). Alexa Fluor555 donkey anti-rabbit IgG (Invitrogen, Karlsruhe, Germany) and goat anti-rat IgG Cy3 antibody (Chemikon, Merck, Darmstadt, Germany) were used as secondary antibodies for further staining. Nuclei were counterstained with DAPI. 

### 2.9. ELISA

The *BMP4^Plt−/−^/LDLr^−/−^* and *BMP4^Plt+/+^/LDLr^−/−^* mice were subjected to carotid wire injury and blood was drawn from retrobulbar sinus at given time points: pre-wire injury and 4 h, 1 day and 7 days after vascular injury. Blood was pooled from 3 animals per experimental group. Pooled blood samples were centrifuged at 2000× *g* for 10 min at 4 °C to obtain serum. The serum concentration of mouse RANTES and PF-4 were measured using mouse RANTES and PF-4 ELISA kits (RayBio Mouse ELISA Kit, Raybiotech, Peachtree Corners, USA) according to the manufacturer‘s protocol.

### 2.10. Flow Cytometry

The cells were washed in PBS, Fc-Receptors were blocked with an antiCD16/CD32 antibody, and the cells were incubated with the indicated antibodies (all from eBioscience, Thermo Fisher Scientific, Waltham, MA, USA, 1:200) before quantification on a flow cytometer (BD FACS Canto II, BD, San Diego, CA, USA). The gating strategy for the monocyte and neutrophil granulocyte populations were based on previous protocol by Leipner et al. [14]. Specifically, the monocyte population was identified as CD45^+^CD11b^+^CD115^+^Ly6C^high/low^ and the platelet-monocyte complex (PMC) as the CD41^+^ subgroup [15,16,17]. The neutrophil granulocyte population was characterized as CD45^+^CD11b^+^CD115^-^Ly6C^intermediate^ based on previous protocol by Leipner et al. [14] and the platelet-neutrophil complex (PNC) as the CD41^+^ subgroup [15,16,17]. For the measurement of levels of P-selectin and active αIIbß3, resting or thrombin activated platelets were incubated with a FITC-labelled anti-P-selectin antibody (BD Bioscience, San Diego, CA, USA) or Oregon Green 488-labelled fibrinogen (Thermo-Fisher Scientific, Karlsruhe, Germany) for 30 min at room temperature. Fluorescence was quantified using FlowJo software (Treestar, Ashland. OR, USA). For each sample, a total of 20,000 events were analyzed. 

### 2.11. Cell Culture

The immortalized mouse cardiac endothelial cells (MCEC) were obtained from Cedarlane (Burlington, Ontario, Canada) at passage 38. Unless otherwise stated, the cells were cultured in DMEM (Gibco, Waltham, MA, USA) without pyruvate containing 10 mM Penicillin/Streptomycin (Gibco, Waltham, MA, USA), 10 mmol/L HEPES (Carl Roth, Karlsruhe, Germany) and 5% FCS (Merck Millipore, Billerica, MA, USA) according to the manufacturer’s instructions and used at passage 42–55. The primary mouse aortic smooth muscle cells were isolated from 4–6 week old mice using a previously described protocol [18]. Bone marrow-derived cells were isolated as previously described [19] and were cultured in DEME supplemented with a 10% bovine growth serum and a 20% L929-conditioned medium. 

### 2.12. In Vitro Cell Proliferation Assay

Cell proliferation was assessed by BrdU incorporation using colorimetric BrdU ELISA assay (Roche, Rotkreuz, Switzerland) according to the manufacturer’s protocol. MCECs (2 × 10^3^ per well) were stimulated by isolated platelets (2 × 10^7^ per well).

### 2.13. Wound Healing Scratch Assay

The migration of MCEC was analyzed by a wound healing scratch assay as described [20]. Briefly, MCECs (2 × 10^5^ per well) were grown to confluency in 6-well plates. Scratch injury was performed by using a pipet tip. Platelets were isolated from murine whole blood and MCECs were stimulated by the isolated platelets (1 × 10^8^ per well) for 24 h. Wound healing was calculated as %wound healing after 6, 12 and 24 h, whereas %wound healing was defined as [(scratch area t0h) − (scratch area tXh)/scratch area t0 h] × 100%.

### 2.14. Western Blot Analysis

The tissue and isolated platelets were homogenized in a RIPA buffer (1 M Tris-HCl pH8, 0.5 M EDTA pH8, 5 M NaCl, Igepal, 20% Na-Deoxycholate, 20% SDS) containing a protease inhibitor cocktail (Calbiochem, Darmstadt, Germany). For Western blot analysis of the carotid arteries, tissues from 3 mice were pooled together. To remove insoluble material, lysates were centrifuged at 10,000× *g* for 10 min at 4 °C and supernatants were collected. The total cellular protein was quantified using Bradford protein assay (Bio-Rad, Munich, Germany) and equal amounts of protein were loaded and separated by a 12% SDS PAGE. Afterwards, the proteins were transferred to nitrocellulose by electroblotting. Western blots were blocked in 3% non-fat dried milk in TBST for 30 min and incubated overnight at 4 °C with primary antibodies against ICAM-1, VCAM-1, and tubulin (BD Bioscience, Heidelberg, Germany). Secondary antibodies used were conjugated with horseradish peroxidase: polyclonal anti-mouse-HRP (R&D Systems/Bio-Techne GmbH, Wiesbaden, Germany), anti-rabbit-HRP (Thermo Fisher Scientific, Darmstadt, Germany), anti-rat-HRP (Dako Deutschland GmbH, Hamburg, Germany). Visualization was performed by ECL (GE Healthcare, Buckinghamshire, UK) and a chemiluminescence detection system (ChemiDoc XRS, Bio-Rad, Munich, Germany). For quantification of the protein band intensities, Image Lab (Bio-Rad, Munich, Germany) was used and expression was normalized to tubulin loading control.

### 2.15. RNA Extraction and Reverse Transcription

DNA-free total RNA was extracted from carotid arteries using the Aurum RNA Mini Kit (Bio-Rad, Munich, Germany). Reverse transcriptions were performed with iScript cDNA-Kit applying 1 µg RNA following the manufacturer’s protocol (Bio-Rad, Munich, Germany).

### 2.16. Quantitative Real-Time PCR

Quantitative PCR analysis was performed using the real-time PCR detection system (Bio-Rad, Munich, Germany) and the MyiQ lightcycler software (Bio-Rad, Munich, Germany). Human RNA-polymerase II was used for normalization. Quantification was calculated using the ΔΔC_T_ method. The following primers were used (Table 1):

### 2.17. Statistical Analysis 

The data were expressed as mean and SEM. Statistical analysis was performed using GraphPad Prism 5.0, USA and comparisons were calculated by Student’s *t*-test, Mann-Whitney *U*-test. A two-way ANOVA was used for repeated measurements followed by Bonferroni´s post-hoc test. A *p* value < 0.05 was considered significant. 

## 3. Results

### 3.1. Basic Characterization of Platelet BMP-4-Deficiency in Mice

Comparative analysis of platelet BMP-4 expression in control (*BMP4^Plt+/+^/LDLr^−/−^*) and *BMP4^Plt−/−^/LDLr^−/−^* mice demonstrated a gene-specific deletion of BMP-4 in platelets (Supplementary Materials File Figure S1A). Phenotypically, there were no differences between the *BMP4^Plt−/−^* and control mice. To determine whether BMP-4 deficiency could lead to defective hemostasis, tail bleeding time was measured in the control and *BMP4^Plt−/−^* mice. There were no discernible differences between the two groups (3.6 ± 0.48 min versus 3.6 ± 0.79 min, Supplementary Materials File Figure S1B). The peripheral blood count between the control and *BMP4^Plt−/−^* mice did not differ (Supplementary Materials File Figure S1C).

### 3.2. Platelet BMP-4 Mediates Neointimal Formation in LDLr^−/−^ Mice

**Figure 1:**
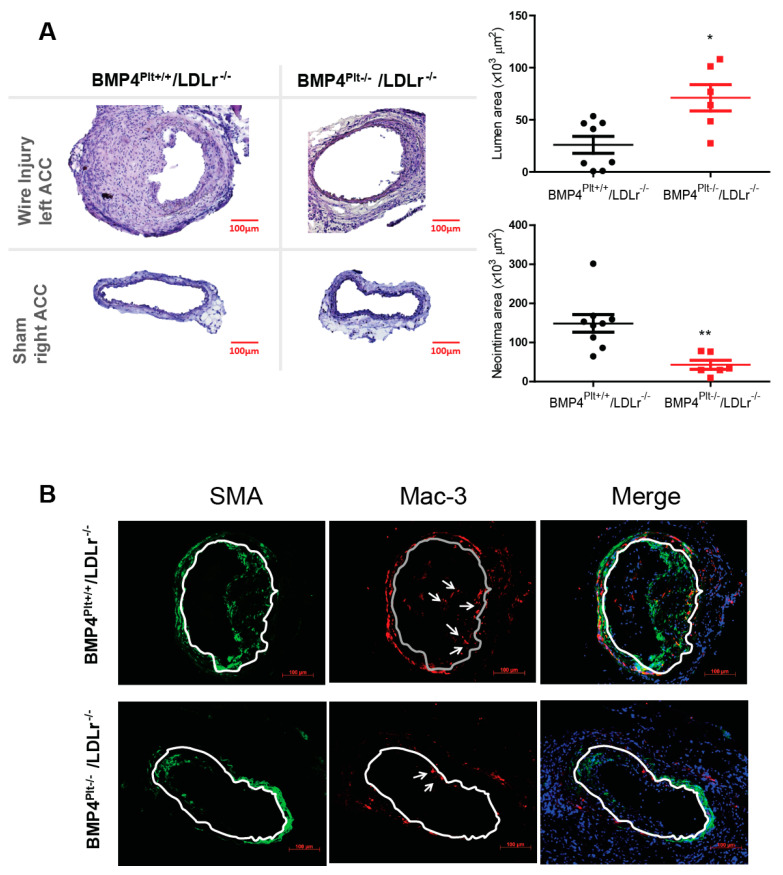
Platelet BMP-4 mediates neointimal formation. (**A**) Representative sections from the carotid arteries after wire injury or sham in BMP4*^Plt−/−^/LDLr^−/−^* mice and control littermates. *n* = 8 for *BMP4^P+/+^/LDLr^−/−^* mice, and *n* = 6 for *BMP4^Plt−/−^/LDLr^−/−^* mice (**B**) Representative images of the carotid lesion stained for SMA and Mac-3. *n* = 3 * *p* ≤ 0.05; ** *p* ≤ 0.01.

Next, we thought to investigate the role of platelet BMP-4 in a disease model. Various reports have suggested a distinct role of platelets in mediating leukocyte recruitment and neointima formation after arterial injury [21,22]. In a real world clinical setting, arterial injury often occurs during vascular angioplasty, and these patients usually have atherosclerosis. Therefore, *BMP4^Ptl+/+^* and *BMP4^Plt−/−^* mice were bred with *LDLr^−/−^* mice to generate an atherosclerosis-prone mouse model. The *BMP4^Plt−/−^/LDLr^−/−^* mice and their littermate control (*BMP4^Plt+/+^/LDLr^−/−^*) at eight weeks of age were fed a Western diet for two weeks, followed by wire injury of the left carotid artery. Two weeks later, the arteries on both sides were excised and analyzed. The size of the neointima lesions was 71.2% larger in the control mice, corresponding to a narrowing of the lumen area by 63.4% compared with the *BMP4^Plt−/−^/LDLr^−/−^* mice (Figure 1A). This finding suggests that the lack of platelet BMP-4 prevented neointimal formation upon injury. Notably, macrophages and smooth muscle cells were significantly decreased in the neointima in the *BMP4^Plt−/−^/LDLr^−/−^* mice compared to control (Figure 1B).

### 3.3. Lack of Platelet BMP-4 Delays Re-Endothelialization by Inhibiting Endothelial Cell Proliferation and Migration

**Figure 2:**
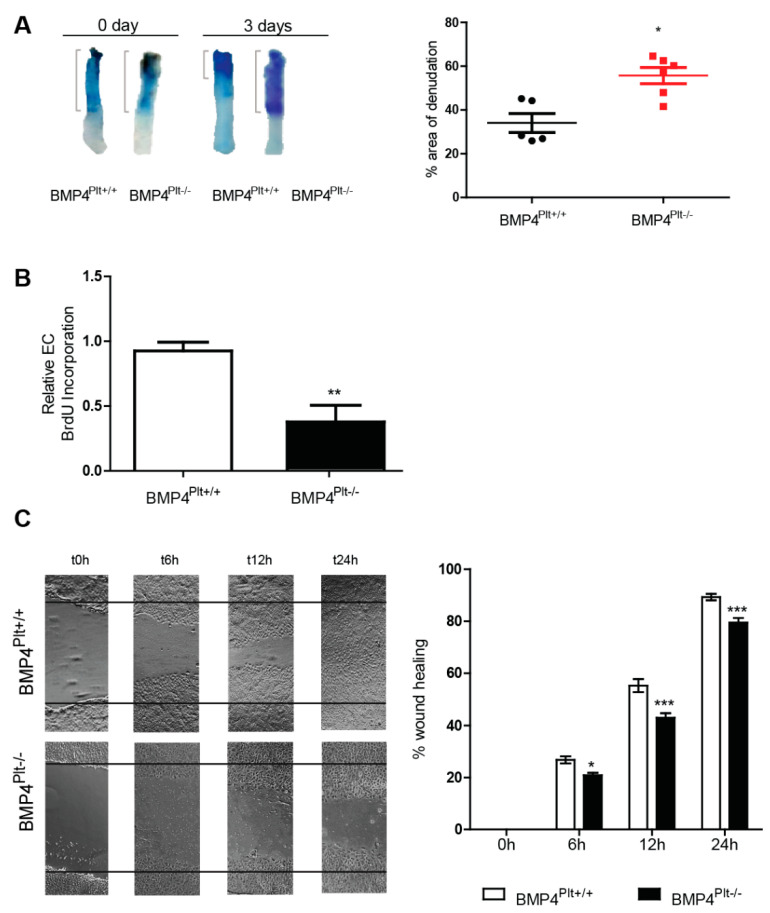
Platelet BMP-4 mediates re-endothelialization, endothelial cell proliferation and migration. (**A**) En-face staining of the denuded carotid with Evans blue after wire injury in *BMP4^Plt−/−^* mice (*n* = 6) and control (*n* = 5). (**B**) Proliferation assay of endothelial cells stimulated with platelets (2 × 10^7^ per well) isolated from *BMP4^Plt−/−^* mice or control littermates. *n* = 5 per group. (**C**) Wound healing of MCECs treated with platelets (1 × 10^8^ per well) isolated from *BMP4^Plt−/−^* mice (*n* = 6 independent experiments) or control littermates (*n* = 5 independent experiments). * *p* ≤ 0.05; ** *p* ≤ 0.01; *** *p* ≤ 0.001.

Re-endothelialization upon vascular injury is an important step in the recovery process and to counter balance reactive neointimal hyperplasia [23,24]. Having demonstrated that neointimal formation was reduced in the *BMP4^Plt−/−^/LDLr^−/−^* mice, we hypothesized that the lack of platelet BMP-4 would also ameliorate the re-endothelialization of the injured carotid artery. Re-endothelialization was quantified by en face Evans blue staining of the denuded area three days after injury. Surprisingly, re-endothelialization was decelerated in the *BMP4^Plt−/−^* mice with a damaged area of 34.1% in control mice, and 55.7% in *BMP4^Plt−/−^* mice at day three (Figure 2A).

Next, we examined whether platelet BMP-4 affects endothelial proliferation and migration in vitro. MCECs were stimulated with platelets isolated from the control or *BMP4^Plt−/−^* mice for 12 h. In line with the in vivo data, the BrdU incorporation assay showed a markedly reduced proliferation in cells stimulated with platelets from the *BMP4^Plt−/−^* mice (Figure 2B). Cell migration is an essential step in the re-endothelialization response. Therefore, we further assessed the effects of platelet BMP-4 on endothelial cell migration using the in vitro scratch-wound assay. In line with the proliferation assay, MCECs were stimulated with platelets isolated from the control or *BMP4^Plt−/−^* mice for 24 h. Wound healing was impaired in the absence of platelet BMP-4. After 6, 12, and 24 h 26.8%, 55.3%, and 89.3% of the wound area recovered in the control mice (Figure 2C). Wound healing in the *BMP4^Plt−/−^* mice were 20.9%, 42.9%, 79.5% after 6, 12, and 24 h, respectively (Figure 2C). In summary, these findings suggest that platelet BMP-4 plays a distinct role in mediating endothelial cell proliferation and migration either directly or indirectly. 

### 3.4. Platelet BMP-4 Mediates Leukocyte Rolling and Adhesion to the Vessel Wall

**Figure 3:**
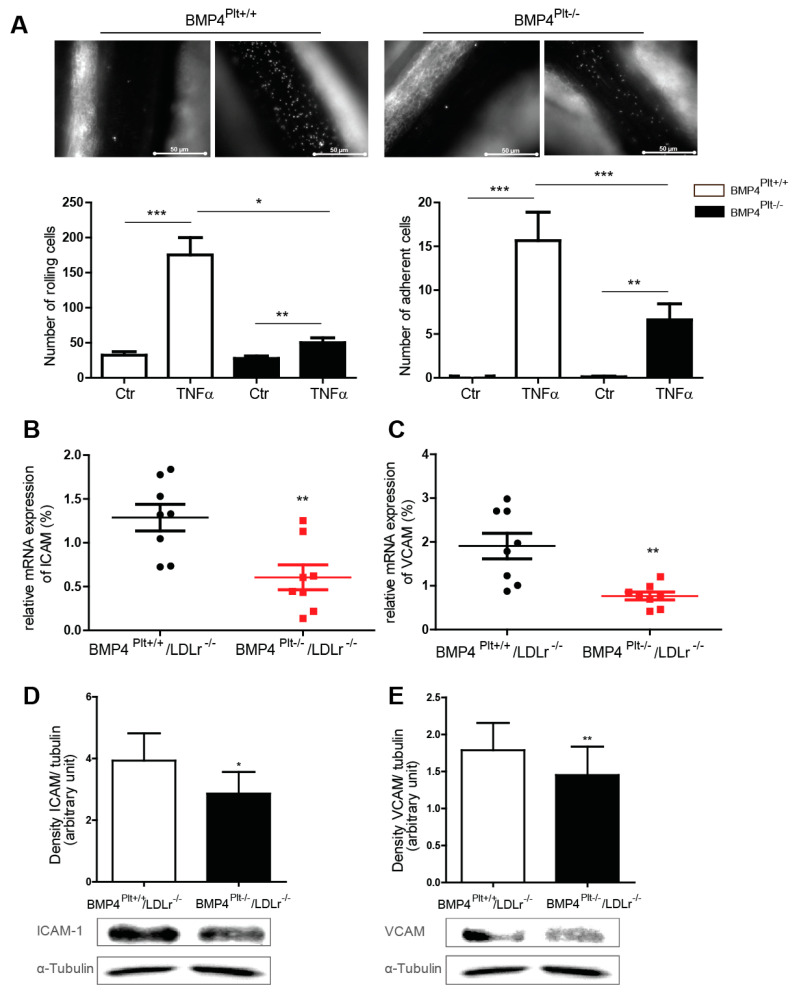
Platelet BMP-4 mediates leukocyte rolling and adhesion to the vessel wall. (**A**) Intravital microscopy of the mesenteric venules of control (*BMP4^Plt+/+^*) or *BMP4^Plt−/−^* mice upon stimulation with TNFα (10 ng/g body weight). *n* = 6 per group. (**B**,**C**) mRNA expression of ICAM-1 and VCAM-1 in carotid arteries from control littermates or *BMP4^Plt−/−^/LDLr^−/−^* mice after guide wire injury. *n* = 8 per group. (**D**,**E**) Protein expression of the adhesion molecules ICAM-1 and VCAM-1 in carotid arteries from control littermates or *BMP4^Plt−/−^/LDLr^−/−^* mice after guide wire injury. *n* = 3 independent experiments; * *p* ≤ 0.05; ** *p* ≤ 0.01; *** *p* ≤ 0.001. n.s., not significant.

Upon vascular injury, platelets are activated and their adhesion to the vessel wall initiates and mediates vascular inflammation. To further evaluate the underlying mechanism, leukocyte interaction with the vessel wall was evaluated using intravital microscopy. The *BMP4^Plt−/−^* mice and control littermates were treated with NaCl or TNFα (10 ng/g body weight), and intravital microscopy was performed after 4 h. In the control mice, treatment with TNFα resulted in a robust increase of rolling cells (175 ± 25 versus 50 ± 7 cells, respectively) and adherent cells (16 ± 3 versus 7 ± 2 cells, respectively), which was markedly reduced in the *BMP4^Plt−/−^* mice (Figure 3A). This finding suggests that the loss of platelet BMP-4 either directly or indirectly affects cell adhesion to the vessel wall, and therefore may play a role in initiating vascular inflammation. 

In a next step, we evaluated how wire injury of the carotid arteries affected vascular inflammation. Two weeks after the guide wire injury, the carotid arteries were isolated, and the expression of ICAM-1 and VCAM-1 were analyzed. In comparison to the control mice, the mRNA level of ICAM-1 and VCAM-1 were significantly less in the injured carotid artery of the *BMP4^Plt−/−^/LDLr^−/−^* mice (Figure 3B,C). This was confirmed at protein level by Western blot (Figure 3D,E). Overall, these findings suggest that platelet BMP-4 is involved in mediating vascular inflammation, potentially through regulating the expression of adhesion molecules in endothelial cells and subsequent interaction of leukocytes with the vessel wall. 

### 3.5. Platelet BMP-4 Is Indispensable for Platelet Activation, Secretion and Aggregation with Leukocytes

**Figure 4:**
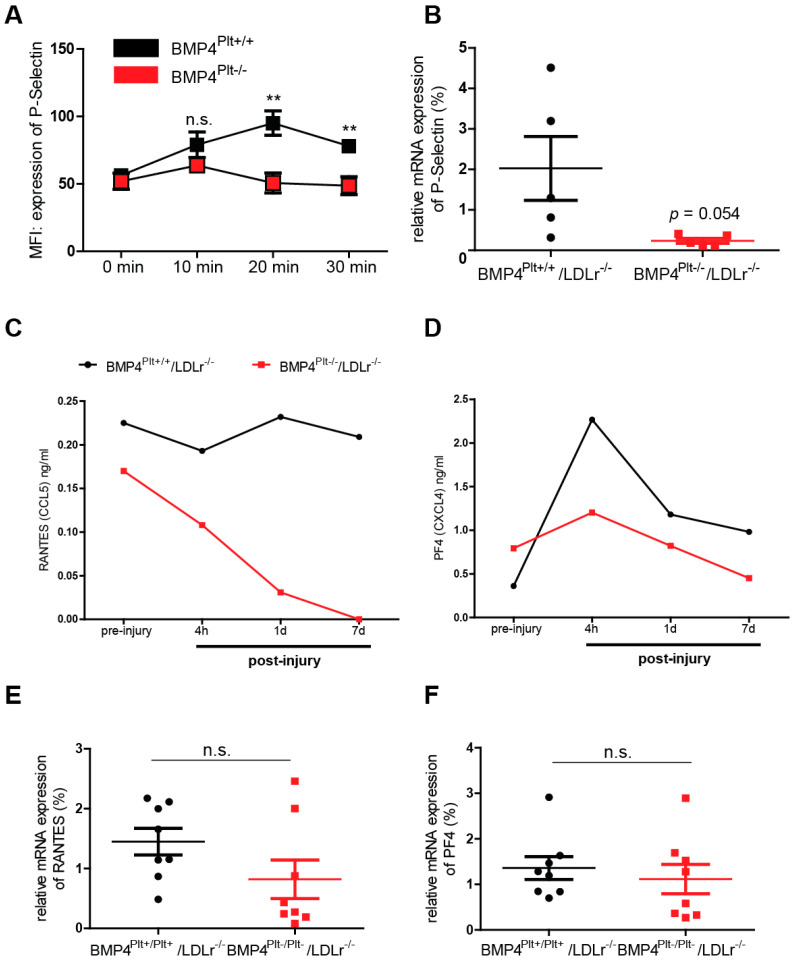
Platelet BMP-4 modulates expression and secretion of P-selectin, RANTES and PF-4. (**A**) Time-dependent release of P-selectin from platelets of control (*BMP4^Plt+/+^*) or *BMP4^Plt−/−^*mice after treatment with TNFα (10 ng/g body weight). *n* = 5 per group. (**B**) mRNA expression of P-selectin in the carotid artery from control or *BMP4^Plt−/−^/LDLr^−/−^* mice after guide wire injury. *n* = 5 per group. (**C**,**D**) Plasma level of RANTES and PF-4 in control littermates or *BMP4^Plt−/−^/LDLr^−/−^* mice after carotid wire injury. Plasma from 3 mice were pooled for each group. (**E**,**F**) mRNA expression of RANTES and PF-4 in the carotid artery of control littermates or *BMP4^Plt−/−^/LDLr^−/−^* mice seven days after guide wire injury. *n* = 8 per group. * *p* ≤ 0.05; ** *p* ≤ 0.01; n.s., not significant.

Activated platelets secret various chemokines, which not only activate the endothelium during vascular inflammation, but also indirectly support leukocyte recruitment via formation of platelet-leukocyte aggregates (PLAs). P-selectin is an important mediator in the formation of PLAs. It is secreted by platelets upon their activation and interacts with the P-selectin glycoprotein ligand (PSGL)-1 on leukocytes [25,26]. To further investigate whether platelet BMP-4 affects the secretion of P-selectin in platelets, the mice were treated with TNFα (10 ng/g body weight) and expression of P-selectin was analyzed using flow cytometry after 10, 20, or 30 min. Interestingly, P-selectin expression increased in a time-dependent manner in the control mice, while its expression level remained unchanged and at a lower level in the *BMP4^Plt−/−^* mice (Figure 4A). The expression of the P-selectin mRNA was also diminished in the *BMP4^Plt−/−^/LDLr^−/−^* mice 14 days after carotid wire injury (Figure 4B), although the result barely missed statistical significance (*p* = 0.054).

In the course of platelet activation, different chemokines are secreted which indirectly contribute to the inflammatory response upon vascular injury. To further evaluate whether platelet BMP-4 would affect chemokine secretion in platelets, serum expression levels of RANTES and PF-4 were measured in the *BMP4^Plt−/−^/LDLr^−/−^* mice and control littermates after carotid wire injury using ELISA. Interestingly, even before injury, the serum level of RANTES was already less in the *BMP4^Plt−/−^/LDLr^−/−^* mice than in the control mice (Figure 4C). Four hours after wire injury, RANTES dramatically decreased in the *BMP4^Plt−/−^/LDLr^−/−^* mice, while it was only slightly reduced in the control mice. After seven days, the carotid arteries were harvested and qRT-PCR was performed. Even though the RANTES mRNA level remained low in the *BMP4^Plt−/−^/LDLr^−/−^* mice, the difference to the control mice was statistically not significant after seven days (Figure 4E). For serum PF-4, the baseline level before injury was slightly higher in the *BMP4^Plt−/−^/LDLr^−/−^* mice. After 4 h the PF-4 level peaked in the control mice, while the serum level remained low in the *BMP4^Plt−/−^/LDLr^−/−^* mice (Figure 4D). After seven days, the expression of PF-4 mRNA in the *BMP4^Plt−/−^/LDLr^−/−^* mice did not differ significantly as compared to the control mice (Figure 4E,F). These findings suggest that RANTES and PF-4 are mostly secreted in the early phase and within the first day after vascular injury, and that its release is partly mediated by platelet BMP-4. 

**Figure 5:**
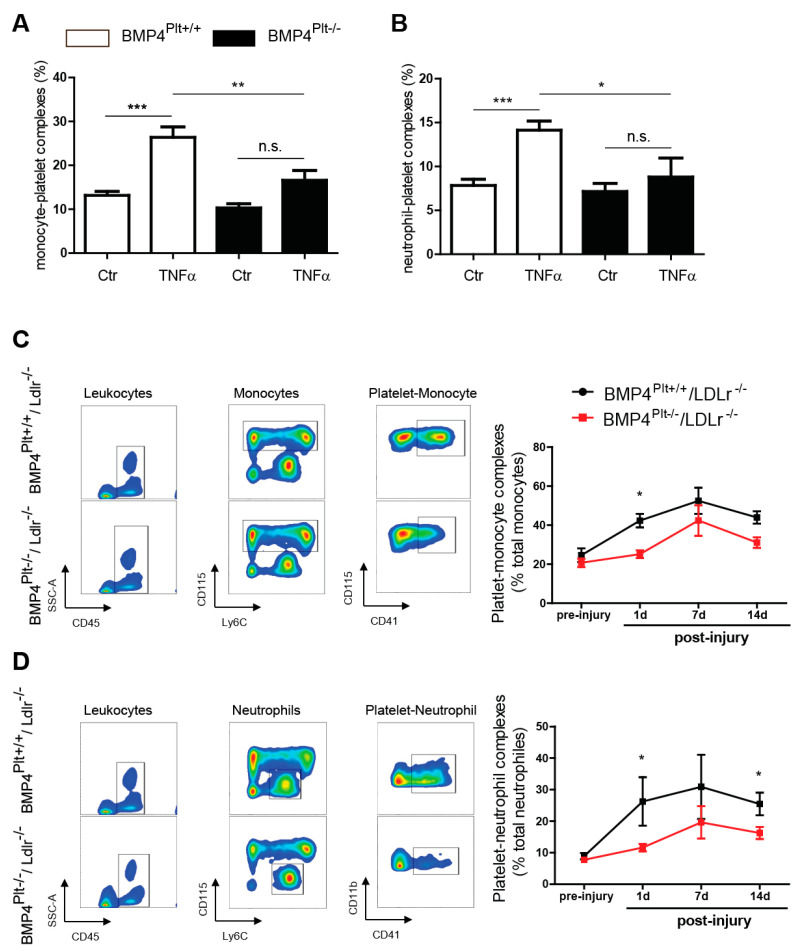
Platelet BMP-4 mediates platelet-leukocyte aggregation. (**A**,**B**) Aggregate formation of platelet-monocyte and platelet-neutrophil in control or *BMP4^Plt−/−^* mice after stimulation with TNFα (10 ng/g body weight), *n* = 6 independent experiments. (**C**) Platelet-monocyte aggregate formation in control littermates or *BMP4^Plt−/−^/LDLr^−/−^* mice after carotid wire injury (*n* = 8 independent experiments). Monocytes were gated from CD45^+^CD11b^+^ cells. (**D**) Platelet-neutrophil aggregate formation in control littermates or *BMP4^Plt−/−^/LDLr^−/−^* mice after carotid wire injury (*n* = 8 independent experiments). Neutrophils were gated from CD45^+^CD11b^+^ cells. * *p* ≤ 0.05; ** *p* ≤ 0.01; *** *p* ≤ 0.001; n.s., not significant.

In a next step, we evaluated the formation of PLAs. Stimulation with TNFα significantly increased the formation of monocyte-platelet aggregates in the control mice, which was less pronounced in the *BMP4^Plt−/−^* mice (Figure 5A). The same result was seen in platelet-neutrophil aggregate formation (Figure 5B). Similarly, platelet-leukocyte aggregation also increased upon guide wire injury of the carotids (Figure 5C,D). The aggregate formation was time-dependent with a peak at day seven after carotid injury and rapidly decreased towards day fourteen. Aggregate formation was more pronounced in the control mice, than in the *BMP4^Plt−/−^/LDLr^−/−^* mice, with the biggest difference between the control and *BMP4^Plt−/−^/LDLr^−/−^* mice at day one. Together, these findings demonstrate that platelet BMP-4 is involved in the regulation of platelet function and the cross-talk of platelets with other immune cells. 

## 4. Discussion

Neointimal hyperplasia is the major cause of restenosis after percutaneous interventions. The underlying molecular mechanisms involve the crosstalk and interplay of various cells, including endothelial cells, smooth muscle cells, leukocytes, and platelets [27]. Indeed, platelet deposition and subsequent leukocyte-platelet interactions on the injured luminal surface are critical in the repair process after arterial damage [28]. 

Previous studies have demonstrated that BMP-4 is upregulated in endothelial cells upon partial ligation of the mouse carotid artery [29,30]. Here, we show for the first time that neointimal hyperplasia is also mediated by BMP-4 in platelets. The loss of platelet BMP-4 prevented neointimal hyperplasia and was associated with less inflammatory response of the injured vasculature.

Our data demonstrate for the first time that platelet BMP-4 affects the expression of adhesion molecules and secretion of chemokines from platelet granules. Stimulation with TNFα in platelets with a loss of BMP-4 was associated with less upregulation of P-selectin than platelets from control mice. P-selectin plays a major role in the adhesion of leukocytes to activated platelets [31,32]. Previous work in chimeric mice that either expressed platelet P-selectin or endothelial P-selectin demonstrated that platelet P-selectin expression, but not endothelial P-selectin, played a crucial role in the development of neointima formation after carotid arterial injury [33]. Through the expression of P-selectin on their surface, activated platelets bind to circulating leukocytes to form PLAs and facilitate the recruitment, rolling, and arrest of monocytes and neutrophils to the activated endothelium [15]. Our study demonstrates that the loss of platelet BMP-4 prevents the formation of PLAs upon exposure to TNFα, and also down-regulates PLAs in *BMP4^Plt−/−^/LDLr^−/−^* mice after carotid artery wire injury. This effect was most pronounced in the early phase after injury with the biggest difference at day one, suggesting that the formation of PLAs is important in the acute phase of inflammatory responses to trigger further immune responses, mediated by chemokines and the interplay of different cell types. 

Besides PLAs, other important key players during vascular injury responses are chemokines [34]. Although platelets are anucleate cells, it is well characterized that they contain diverse chemokines and growth factors stored in granules [35]. The secretion of these granules is important in orchestrating further inflammatory responses, e.g., PLA formation, or heterotypic interactions of platelets with other inflammatory cells. Indeed, our study demonstrates that the loss of platelet BMP-4 is associated with reduced secretion of RANTES and PF-4 from platelets and their deposition at the sites of injury. RANTES and PF-4 have been well characterized for their roles in mediating vascular inflammation [4,5]. RANTES has been shown to bind to injured endothelium where it enhances monocytes recruitment and activates monocyte integrins [36]. In line with this report, our study showed fewer rolling and adherent cells in the *BMP4^Plt−/−^* mice upon stimulation with TNFα, than in the control mice. This finding was further supported by the reduced expression of ICAM and VCAM in the carotid arteries of the *BMP4^Plt−/−^/LDLr^−/−^* mice. PF-4, on the other hand, has been shown to trigger the activation and adhesion of neutrophils and monocytes to endothelial cells [6]. Additionally, it has been reported that PF-4 accelerated inflammatory responses of vascular smooth muscle cells after vascular injury. In comparison to the control mice, the PF4^−/−^ mice developed less neointimal hyperplasia after carotid ligation [37]. This finding could partially explain our observation that neointimal hyperplasia was less pronounced in the *BMP4^Plt−/−^/LDLr^−/−^* mice than in the control mice, as the loss of platelet BMP-4 was also associated with the reduced secretion of PF-4 after vascular injury. 

Surprisingly, although neointimal formation was markedly reduced in the *BMP4^Plt−/−^/LDLr^−/−^* mice, the regeneration of the surface endothelium was decelerated in these mice. In line with the in vivo findings, in vitro studies using MCECs demonstrated an inhibition of cell proliferation and migration when stimulated with activated platelets lacking BMP-4. Similar results have been previously described for BMP-4 and smooth muscle cells. Using heterozygous BMP-4 (*lacZ/+*) mice, Corriere et al. demonstrated that BMP-4 was upregulated in endothelial cells after ligation of the carotid artery [30]. However, when smooth muscle cells were stimulated with human recombinant BMP-4 in vitro, cell migration was decelerated, but not SMC proliferation. The authors concluded that BMPs may counterbalance the effect of mitogen up-regulation occurring during the development of neointimal hyperplasia [30]. Another explanation for this finding could be the role of inflammation during vascular remodeling per se. Previous studies have demonstrated that inflammation and angiogenesis, a process involving endothelial cell proliferation, are linked to each other [38] and balanced by angiopoetins-1 and -2. Additionally, circulating leukocytes and platelets which are recruited during inflammation have been shown to produce large quantities of pro-angiogenic factors, including VEGF and cytokines [39,40]. Furthermore, stimulation of RANTES was associated with increased neovessel formation in an in vivo disc angiogenesis in rats, and increased endothelial migration and sprouting [41]. As RANTES secretion was less pronounced in platelets with a loss of BMP-4, this finding could partly explain our observation that endothelial migration was less pronounced after stimulation with BMP-4 deficient platelets. Overall, while the loss of platelet BMP-4 reduces inflammatory responses, its inhibitory effect on leukocyte recruitment and cytokine secretion may impair endothelial cell proliferation and eventually angiogenesis. 

## 5. Conclusions

In the past years, many efforts have been made to understand the molecular mechanisms of vascular remodeling upon injury. In our present study, we show that platelets, in addition to their well-known function in hemostasis and thrombosis, regulate the inflammatory response during vascular remodeling by facilitating platelet-leukocyte interaction, leukocyte migration and adhesion to the vessel wall. Loss of platelet BMP-4 was associated with reduced platelet activation and reduced formation of PLAs, which could play important roles in the initiation of inflammatory response. Subsequent effects of platelet BMP-4 on the secretion of chemokines further triggered the inflammatory vascular remodeling. Our findings uncover a previously unrecognized function of BMP-4 in platelets. However, several questions remain, for example, the specific effect of platelet BMP-4 on endothelial cells, vascular smooth muscle cells and leukocytes during vascular injury. In addition, it is unclear whether the protective effect of platelet BMP-4 is only limited to the early phase of acute vascular injury or has a long lasting effect in chronic vascular inflammation, e.g., atherosclerosis. Further studies are needed to address the specific function of the BMP signaling pathway in platelets. Moreover, it remains to be determined whether the inhibition of platelet BMP-4 will have therapeutic benefits in patients undergoing percutaneous interventions. Nevertheless, this study provides first evidence regarding the importance of platelet BMP-4 in vascular inflammation and remodeling.

## Figures and Tables

**Table 1:** List of primers.

Gene	Forward (5′-3′)	Reverse (5′-3′)
*HRP II*	GCA CCA CGC CAA TGA CAT	GTG CGG CTG CTT CCA TAA
*ICAM*	AGG TGG TTC TTC TGA GCG GC	AAA CAG GAA CTT TCC CGC CA
*VCAM*	TCT TGG GAG CCT CAA CGG TA	CAA GTG AGG GCC ATG GAG TC
*RANTES*	GCA AGT GCT CCA ATC TTG CA	CTT GGC GGT TCC TTC GAG T
*PF-4*	GAG GTG ATC AAG GCA GGA CG	TAT AGG GGT GCT TGC CGG TC
*P-selectin*	CCT GGC AAG TGG AAT GAT GA	AAG CTG CAG ACT GAC TGG TA

## Data Availability

Data are available upon reasonable request from the corresponding author. Reagents and detailed methods of all procedures are provided in "Material and Methods” of this manuscript.

## Supplementary Materials

The following are available online at https://www.mdpi.com/article/10.3390/cells10082027/s1, Figure S1: Basic characterization of BMP4^Plt−/−^ mice.
