# Supplementary material for: Platelet Bone Morphogenetic Protein-4 Mediates Vascular Inflammation and Neointima Formation after Arterial Injury
Source: Cells. 2021 Aug 8;10(8):2027. doi: 10.3390/cells10082027 (PMC8394465; doi:10.3390/cells10082027)
Supplement: Supplementary file 1 [file cells-10-02027-s001.zip › cells-1264684-supplementary.pdf]

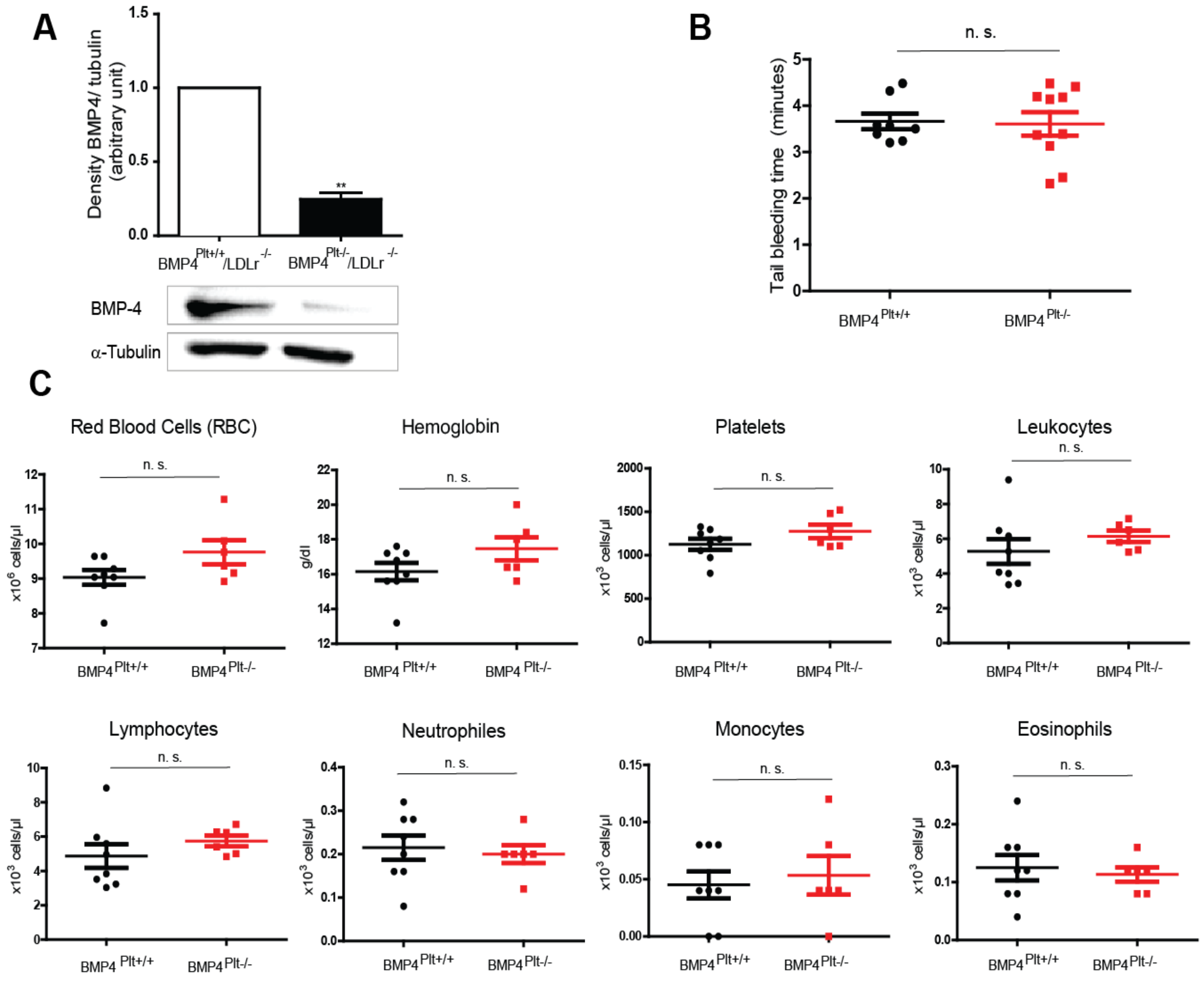

Figure S1. Basic characterization of BMP$^{Plt-/-}$ mice. (**A**) Protein expressionof BMP-4 in platelets from BMP$^{Plt+/+}$/LDLr$^{-/-}$ mice. (**B**) Bleeding times of control (BMP$^{Plt+/+}$) and BMP$^{Plt-/-}$ mice. (**C**) Peripheral blood count between BMP$^{Plt+/+}$ (control) and BMP$^{Plt-/-}$ mice. n = 3 independent experiments for (**B**). ** $p \leq 0.01$; n.s., not significant.
